# Maybe she's NOT the boss: male–female crosstalk during sexual plant reproduction

**DOI:** 10.1186/s13059-016-0972-6

**Published:** 2016-05-09

**Authors:** Hannes Vogler, Andrea Martinez-Bernardini, Ueli Grossniklaus

**Affiliations:** Department of Plant and Microbial Biology and Zurich-Basel Plant Science Center, University of Zurich, Zollikerstrasse 107, 8008 Zurich, Switzerland

## Abstract

New insights into the molecular dialogue between male and female during sexual plant reproduction show that even plant sex does not work without clear communication.

Please see related Research article: http://genomebiology.biomedcentral.com/articles/10.1186/s13059-016-0928-x

Plants provide us with almost all of the food, feed, fuel, and fibers we need. Ninety percent of our daily energy intake comes directly or indirectly from flowering plants, the majority of which reproduce sexually. Even species that are able to propagate vegetatively usually retain the ability to remix their genomic composition by meiotic recombination, i.e., through sex. Classic plant breeding that results in the creation of new genetic combinations would be unthinkable without the recombination of specific traits by crossing male and female plants harboring them, followed by selection of the desired characteristics. Sexual reproduction in plants requires extensive cell–cell communication between male and female gametes. In a recent article in *Genome Biology*, Hafidh and colleagues unravel some of the components involved in this process [1].

## How do immotile sperm cells find the female gametes in flowering plants?

**Fig. 1:**
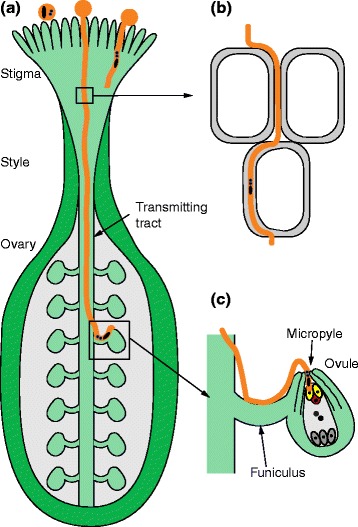
Pollination in sexual plant reproduction. **a** Overview of a pollinated pistil: pollen grains (*orange circles*) land on the stigma. Compatible pollen grains hydrate and germinate and the pollen tubes (*orange*) grow within the transmitting tract through the style towards the ovary to deliver the sperm cells to the ovules. The two sperm cells (*blue*) are contained in a common extracellular matrix within the pollen tube and connected to the vegetative nucleus (*black ellipse*) by a cytoplasmic projection. **b** In the transmitting tract, pollen tubes can grow between neighboring cells or penetrate a cell and grow within the cell wall. **c** The pollen tube, after exiting the transmitting tract and being attracted by an unfertilized ovule, grows along the funiculus to the micropyle. After appropriate signaling during pollen tube reception, the pollen tube grows rapidly across and penetrates one of the two synergids (*yellow*), ruptures, and releases the two sperm cells, one each fertilizing the egg cell (*red*) and the central cell (*gray*) to produce the embryo and the endosperm, respectively

In flowering plants, gametes are produced by male and female gametophytes that develop within the sexual organs of the flower. The meiotic products divide to form male (pollen) and female (embryo sac) gametophytes. The embryo sac, containing the two female gametes participating in double fertilization, is harbored inside an ovule, which is the precursor of the seed. Pollen consists of two immotile sperm cells, which are carried within the cytoplasm of a large vegetative cell. An amazing aspect of sexual reproduction in flowering plants is that a tiny pollen grain, with a diameter of a few tens of microns, can give rise to a pollen tube that grows to several hundred times that length. The pollen tube, formed by the vegetative cell, elongates at an incredible speed of up to 1 cm per hour, eventually delivering the two sperm cells to the embryo sac, fertilizing the two female gametes during double fertilization (Fig. 1). Pollen tubes have a highly optimized growth machinery, allowing them to quickly reach one of the ovules within the female reproductive tissue, which is often several centimeters away from the stigma where the pollen grain has germinated. Pollen tubes that grow too slowly, or cannot properly find their way, are lost to evolution as they do not contribute to the next generation.

The path from the stigma to the ovules is not only long but also complex as diverse tissues have to be navigated. Thus, the pollen tube has to sense the location of the ovules and precisely find the micropyle, an opening that is only a little wider than its own diameter. A whole cascade of guidance signals is launched from both partners to make sure that only compatible pollen grains germinate and grow a pollen tube. Once on its way, the pollen tube is guided through the transmitting tract (sporophytic guidance) by a plethora of secreted proteins, peptides, and chemoattractants [2]. Eventually, after exiting the transmitting tract, another set of signaling molecules lures the pollen tube along the funiculus to the micropyle. After an extended foreplay, which can last for half an hour and is characterized by a male–female dialogue mediated by calcium [3], the pollen tube finally penetrates an accessory cell of the embryo sac, bursts, and releases the sperm cells enabling double fertilization. One sperm fertilizes the egg to produce the zygote and eventually the embryo, while the other fuses with the central cell to form the endosperm, which provides nutritional resources to the developing embryo.

Over recent years, researchers have invested a lot of energy in unraveling the secrets of male–female crosstalk during sexual plant reproduction. However, while a fair number of female signaling molecules have been identified [4, 5], very few male contributors to this dialogue are known. Given the predominance of known female factors, it is not surprising that “she“ was considered the boss, controlling pollen tube behavior [6]. Now, Hafidh and colleagues have undertaken the effort to analyze the first pollen tube secretome and shed some light on how its composition changes through interaction with female reproductive tissues [1].

## Why did it take so long to isolate the pollen tube secretome?

Pollen tubes growing in the transmitting tissue are extremely difficult to access and separate from the cells of female tissues. Imbibing pollen in germination medium and growing the pollen tubes in vitro circumvents these difficulties but prevents contact between pollen tubes and female tissues. The solution for these problems came in the form of a semi-in vivo approach. After pollination of intact flowers, the pollen tubes were allowed to grow out of cut styles that were incubated in pollen tube growth medium. Proteins that were secreted from the emerging pollen tubes into the medium were then further analyzed by liquid chromatography followed by tandem mass spectrometry (LC-MS/MS). In comparison with the secretome of in vitro-grown pollen tubes, the secreted molecules obtained using this approach are induced by the female reproductive tissues. Thus, they can accurately detect those male actors that participate in male–female interactions. A similar approach has recently been used in order to determine the ovule secretome [5].

## Unconventional protein secretion plays a role in the male–female crosstalk during plant reproduction

Hafidh and colleagues identified a series of proteins with potential function in male–female interactions [1]. The authors performed a meticulous in silico study, providing valuable predictions about the function, trafficking, and other characteristics of the identified proteins. Many of them were confirmed as actors in the male–female dialogue, while some of them seem to be novel players. Curiously, a significant percentage of “unconventional protein secretion” (UPS) was observed. These proteins lack the typical N-terminal signal peptide which determines secretion through the endoplasmic reticulum pathway.

While the presence of UPS in plants is generally accepted, direct evidence for the translocation of such proteins through the plasma membrane was scarce. In fact, there has been only one verified case confirming the extracellular localization of Helja, an unconventionally secreted sunflower protein [7]. With a careful colocalization study, Hafidh and colleagues not only confirmed conventional trafficking, as well as the decisive role of secretory components regulating it, but they also provided evidence for UPS at work. They show that the model protein translationally controlled tumour protein (TCTP) is potentially transported through the initial phases of the secretory pathway but then crosses the plasma membrane via exosomes. This depicts a new mechanism of cell–cell communication in the context of sexual plant reproduction, possibly involved in long distance signaling in the male–female crosstalk.

## Many secreted proteins are post-transcriptionally regulated during pollen tube growth

A comparison of the tobacco pollen tube secretome with the transcriptome of semi-in vivo-grown pollen tubes of *Arabidopsis* revealed some interesting findings. Notably, the two data sets show a near-complete qualitative overlap but the relative abundance of secreted proteins is not linearly correlated with transcript levels. While cross-species comparisons have their own caveats, these data nonetheless suggest that secreted proteins are subject to post-transcriptional regulation. This is not an isolated case as it has previously been observed in the ovule secretome of *Solanum chacoense* [5] as well as in other organisms. Therefore, the widely applied transcriptomics approaches clearly have limitations, particularly with respect to secreted proteins, and should be combined with complementary approaches to obtain a more complete picture of the process under study.

With their recent study, Hafidh and colleagues open the field for a more detailed investigation of the molecular dialogue taking place during sexual plant reproduction [1]. The effects of specific female guidance molecules on the secretome of pollen tubes can be assessed by the in vitro application of such molecules or by using the styles of mutant plants that are lacking certain guidance molecules in a semi-in vivo approach. Obviously, this would also work to study the secretome of the embryo sac and other female tissues by exploiting a similar method as described by Liu and colleagues [5].

## Does sexual plant reproduction have something to do with plant defense?

In a seemingly different field of research, there is growing evidence that pathogen-related proteins are secreted via UPS. However, pathogen attacks may not be all that different from the crosstalk during fertilization with respect to biochemical signaling. *Arabidopsis* plants lacking FERONIA, a receptor-like kinase that is involved in pollen tube reception at the ovule, show resistance against powdery mildew, a well-known fungal pathogen of plants [8]. Moreover, FERONIA also plays a role in innate immunity and mutants show an enhanced production of reactive oxygen species and reduced bacterial proliferation upon infection [9]. It will be interesting to uncover how far the parallels between plant–pathogen and male–female interactions go.

## Abbreviation

UPS: unconventional protein secretion
